# Cytokine imbalance and HBV-specific T-cell exhaustion predict disease progression in HIV-HBV coinfection

**DOI:** 10.3389/fimmu.2026.1789692

**Published:** 2026-03-04

**Authors:** Peter Asaga Mac, Dave C. Ibeh, Mansur Aliyu Ramalan, Bako A. Ishaku

**Affiliations:** 1 Institute of Infectious Disease Control and Hospital Epidemiology, Freiburg, Germany; 2 NIL, Nigeria Army Reference Hospital, Sokoto, Nigeria; 3 Department of Clinical Pharmacology, Federal University of Lafia, Lafia, Nigeria; 4 Department of Epidemiology, Federal University of Lafia, Lafia, Nigeria

**Keywords:** cytokine dysregulation, HBV reactivation, HIV-HBV coinfection, liver fibrosis, Nigeria, T-cell exhaustion

## Abstract

**Background:**

HIV-HBV coinfection accelerates liver disease, yet the immunological mechanisms underlying adverse outcomes remain incompletely characterized in African populations. We investigated relationships between HBV reactivation, cytokine dysregulation, T-cell dysfunction, and disease progression in a Nigerian cohort.

**Methods:**

We screened 1,139 participants across four Nigerian states. Of 344 HIV-positive individuals, 53 (15.4%) had HBV coinfection. For detailed immunological and longitudinal analyses, 59 coinfected participants with complete datasets were included in the mechanistic cohort. Comprehensive assessments including HBV DNA quantification, S-gene sequencing, cytokine profiling, and HBV-specific T-cell responses were performed on 59 coinfected patients with longitudinal follow-up.

**Results:**

Phylogenetic analysis indicated 71.4% (30/42) of cases with rising HBV DNA were consistent with reactivation. HBV genotype E predominated (94.3%). Coinfected patients demonstrated elevated IL-6 and TNF-α with reduced IFN-γ compared with HIV-monoinfected controls (all p < 0.001). The IL-6/IFN-γ ratio correlated with HBV viral load (r = 0.74), APRI score (r = 0.71), and CD4+ count (r = −0.64; all p < 0.001). HBV-specific polyfunctional CD8+ T-cells were markedly reduced (median 0.08% vs 3.8% in controls; p < 0.001). In multivariable Cox regression, IL-6/IFN-γ ratio >4.0 (HR 4.12, 95% CI 1.86–9.14), CD4+ <200 cells/µL (HR 3.24, 95% CI 1.58–6.64), and APRI >1.0 (HR 2.86, 95% CI 1.34–6.11) independently predicted progression, whilst preserved T-cell polyfunctionality was protective (HR 0.32, 95% CI 0.15–0.68).

**Conclusions:**

HIV-HBV coinfection was characterized by HBV reactivation, cytokine imbalance, and T-cell exhaustion, which were associated with disease progression and may inform risk stratification.

## Introduction

Hepatitis B virus (HBV) infection affects approximately 296 million people globally and remains a leading cause of cirrhosis and hepatocellular carcinoma (1). Among people living with HIV, HBV coinfection occurs in 5–20% depending on geographical region and is associated with accelerated liver disease progression and increased mortality (2–4). Sub-Saharan Africa bears a disproportionate burden of both infections, with Nigeria alone accounting for substantial numbers of coinfected individuals (5, 6).

HIV-induced immunodeficiency fundamentally alters the host-HBV relationship. CD4+ T-cell depletion impairs adaptive immune responses essential for HBV control, whilst chronic immune activation promotes hepatic inflammation and fibrogenesis (7, 8). Individuals with prior HBV exposure who achieve apparent viral control may experience reactivation during immunosuppression, with potentially severe clinical consequences (9). However, the specific immunological signatures that distinguish patients at highest risk of HBV reactivation and disease progression remain incompletely defined, particularly in African populations where HBV genotype E predominates (10).

Cytokines coordinate antiviral immunity and mediate liver inflammation. Interleukin-6 (IL-6) and tumor necrosis factor-alpha (TNF-α) are pro-inflammatory mediators implicated in hepatic fibrosis, whilst interferon-gamma (IFN-γ) is essential for non-cytolytic viral clearance (11, 12). The balance between these responses may determine clinical outcomes. Additionally, virus-specific T-cell function, particularly polyfunctional responses capable of simultaneous cytokine production, correlates with pathogen control across multiple infections (13). In chronic HBV monoinfection, T-cell exhaustion characterized by elevated programmed death-1 (PD-1) expression and reduced functionality contributes to viral persistence (14, 15).

We conducted a prospective cohort study to characterize HBV reactivation patterns, cytokine profiles, and virus-specific T-cell responses in Nigerian patients with HIV-HBV coinfection, and to evaluate associations between these parameters and disease progression.

## Methods

We conducted a prospective cohort study screening 1,139 participants across four Nigerian states between January 2021 and December 2024 to identify individuals with HIV–HBV coinfection and to characterize HBV reactivation patterns, cytokine profiles, virus-specific T-cell responses, and their associations with disease progression. The screened population comprised 1,139 individuals. Of the 1,139 participants screened, 344 were HIV-positive, and 53 of these had confirmed HBV coinfection based on HBsAg positivity. For the mechanistic substudy, additional eligible participants with stored longitudinal samples and complete datasets were included, resulting in a final analytical cohort of 59 HIV–HBV.

Participants were recruited from HIV treatment centers and voluntary counselling and testing sites using stratified sampling to ensure geographic representation. All participants underwent HIV testing, and those confirmed HIV-positive were subsequently tested for HBV coinfection. Of the 1,139 participants screened, 344 (30.2%) were HIV-positive, and of these, 53 (15.4%) had confirmed HBV coinfection based on hepatitis B surface antigen (HBsAg) positivity. For the mechanistic substudy, additional eligible participants with stored longitudinal samples and complete datasets were included, resulting in a final analytical cohort of 59 HIV–HBV coinfected individuals.

For the mechanistic substudy, inclusion criteria comprised age ≥18 years, confirmed HIV-HBV coinfection, and adequate sample volume for comprehensive virological and immunological analyses. Exclusion criteria included pregnancy, active opportunistic infections requiring hospitalization, hepatitis C or D coinfection, and hepatocellular carcinoma at baseline. Following application of eligibility criteria and inclusion of longitudinal follow-up samples, 59 HIV-HBV coinfected patients were included in the final analysis (**Supplementary Figure 1**).

A comparison group of 59 HIV-monoinfected patients matched for age, sex, and CD4+ count was enrolled from the same centers. Healthy controls (n = 30) for T-cell functional assays were recruited from HBV-vaccinated, HIV-negative blood donors matched for age and sex.

### Laboratory methods

HIV infection was confirmed using sequential rapid diagnostic tests with enzyme-linked immunosorbent assay (ELISA) confirmation per Nigerian national algorithms. HBV infection was diagnosed using Monolisa HBsAg ULTRA (Bio-Rad Laboratories) and Determine HBsAg rapid tests (Abbott Diagnostics). Plasma HBV DNA was quantified using COBAS AmpliPrep/COBAS TaqMan HBV Test version 2.0 (Roche Diagnostics; lower limit of detection 20 IU/mL). CD4+ T-cell counts were measured by flow cytometry (16).

Plasma cytokines (IL-6, TNF-α, IFN-γ) were quantified using Bio-Plex Pro Human Cytokine Assay (Bio-Rad Laboratories) in duplicate with internal quality controls. The IL-6/IFN-γ ratio was calculated as a measure of pro-inflammatory versus antiviral balance (17).

HBV-specific CD8+ T-cell responses were assessed by intracellular cytokine staining following stimulation with overlapping HBV core and surface antigen peptide pools. Polyfunctional T-cells were defined as those producing ≥2 cytokines (IFN-γ, TNF-α, IL-2) simultaneously. PD-1 expression was quantified as a marker of T-cell exhaustion (18). Healthy controls (n = 30) were recruited from HBV-vaccinated, HIV-negative blood donors matched for age and sex.

### HBV sequencing and phylogenetic analysis

Partial S-gene sequencing (~680 bp) was performed in patients with available paired baseline and follow-up samples. Sequences were aligned with HBV genotype reference sequences obtained from GenBank (**Supplementary Table 1**), including genotypes A, D, and E, using ClustalW (19). Maximum-likelihood phylogenetic trees were constructed using the GTR+Γ substitution model with midpoint rooting in MEGA version 11.0 (20). Genotype assignment required bootstrap values >70% and sequence identity >92% with reference sequences.

To distinguish reactivation from new infection, pairwise genetic distances between baseline and follow-up sequences from each patient were calculated. Based on established HBV evolutionary rates, genetic distances <2% between timepoints were considered consistent with reactivation of persistent infection, whilst distances >4% suggested new infection acquisition (21). Intermediate distances were classified as indeterminate.

### Liver fibrosis assessment

Liver fibrosis was assessed using the aspartate aminotransferase-to-platelet ratio index (APRI), calculated as (AST/upper limit of normal × 100)/platelet count (×10^9^/L). APRI >1.0 indicated significant fibrosis; >2.0 suggested cirrhosis (22).

### Outcomes

The primary outcome was disease progression, defined as a composite of hepatic decompensation, progression to WHO clinical stage 4, new AIDS-defining illness, or death. Participants were followed for up to 24 months.

### Statistical analysis

Continuous variables are presented as median (interquartile range [IQR]) or mean ± standard deviation. Group comparisons used Mann-Whitney U or Kruskal-Wallis tests. Correlations were assessed using Spearman coefficients. Kaplan-Meier curves estimated progression-free survival, compared by log-rank tests. Cox proportional hazards regression identified predictors of progression; the proportional hazards assumption was verified using Schoenfeld residuals. Model discrimination was assessed using Harrell’s C-statistic (23). Analyses were performed using Stata version 17.0 and R version 4.2.0, with p < 0.05 considered significant.

### Ethical approval

This study was conducted in accordance with the Declaration of Helsinki and approved by the Ethics Committee of the University of Freiburg, Germany (approval number 140/19) and the National Health Research Ethics Committee of Nigeria (NHREC/01/022). Written informed consent was obtained from all participants prior to enrolment. Participant confidentiality and data protection were strictly maintained throughout the study.

## Results

### Study population

A total of 1,139 participants were screened across four Nigerian states, of whom 344 (30.2%) were HIV-positive. HBV coinfection was present in 53 of the 344 HIV-positive individuals (15.4%), yielding an overall HIV-HBV coinfection prevalence of 4.7% (53/1,139) in the screened population. Following application of eligibility criteria and inclusion of longitudinal samples, 59 HIV-HBV coinfected patients were included in the mechanistic analysis (**Supplementary Figure 1**).

Baseline characteristics are shown in **Table 1**. Median age was 36 years (IQR 29–44), 54.2% were female, and median CD4+ count was 267 cells/µL (IQR 156–342). 25 (42.4%) participants had CD4+ counts <200 cells/µL. Geographic distribution included Abia (n = 21, 35.6%), Kaduna (n = 21, 35.6%), Nasarawa (n = 19, 32.2%), and Benue (n = 16, 27.1%) states.

**Table 1:** Baseline characteristics of HIV-HBV coinfected patients (n = 59).

Characteristic	Value
Age, years, median (IQR)	36 (29–44)
Female sex, n (%)	32 (54.2)
CD4+ count, cells/µL, median (IQR)	267 (156–342)
CD4+ <200 cells/µL, n (%)	25 (42.4)
HBV DNA, log_10_ IU/mL, median (IQR)	3.9 (2.8–4.8)
ALT, U/L, median (IQR)	68 (42–99)
APRI score, median (IQR)	1.24 (0.68–2.14)
APRI >1.0, n (%)	34 (57.6)

IQR, interquartile range; ALT, alanine aminotransferase; APRI, aspartate aminotransferase-to-platelet ratio index.

### HBV genotyping and reactivation analysis

**Figure 1:**
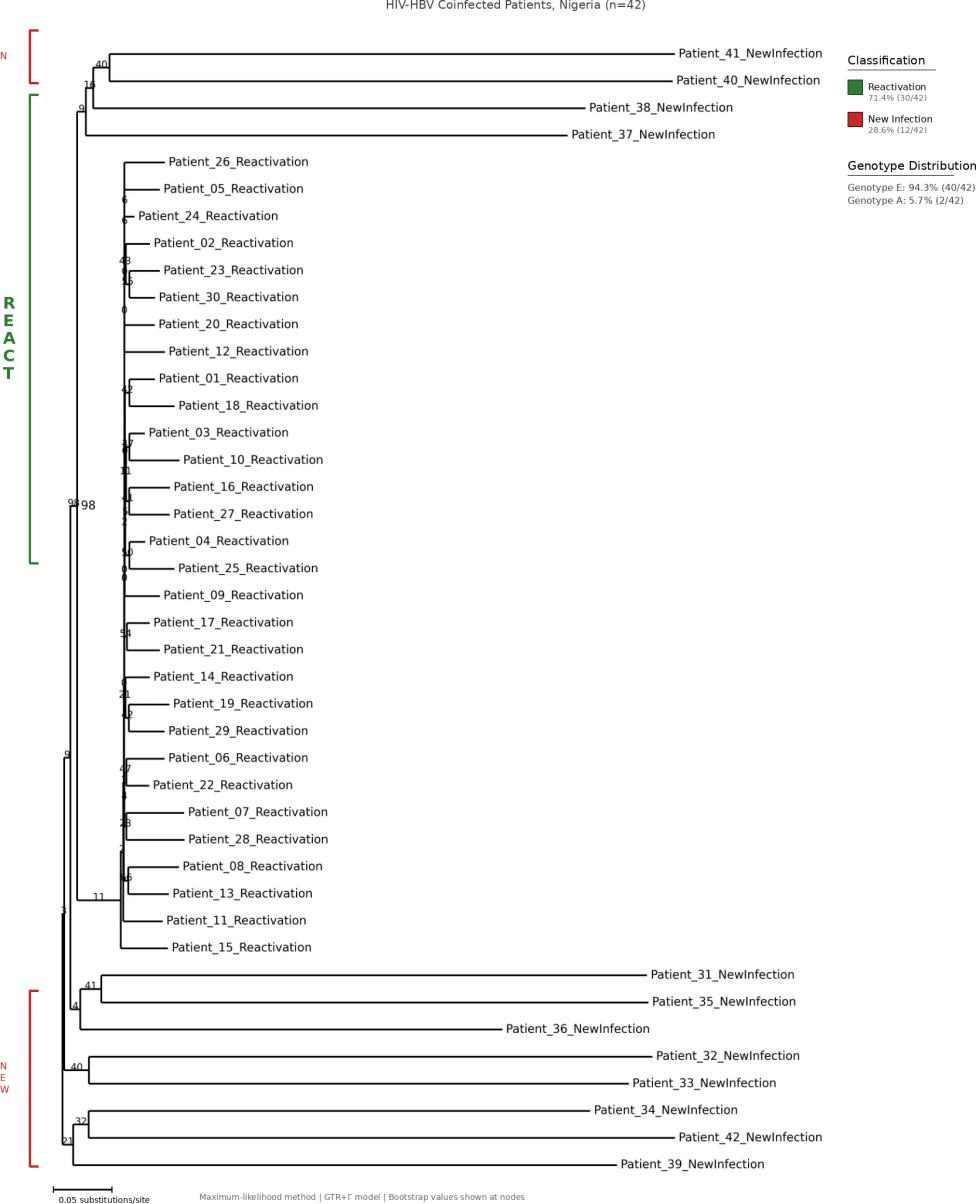
Phylogenetic analysis of HBV sequences from HIV-HBV coinfected patients. Maximum-likelihood cladogram of partial S-gene sequences (~680 bp) from 42 patients using GTR+Γ model with midpoint rooting. Genotype E predominated (94.3%). Based on sequence divergence criteria, 71.4% (30/42) were classified as reactivation and 28.6% (12/42) as new infection.

Phylogenetic analysis was performed on 42 patients with paired baseline and follow-up sequences. HBV genotype E predominated (94.3%, 40/42), with genotype A comprising the remainder (5.7%, 2/42), consistent with West African epidemiology (10). Based on sequence divergence criteria, 71.4% (30/42) of cases with rising HBV DNA level (viral load) were classified as consistent with viral reactivation, whilst 28.6% (12/42) showed patterns consistent with new infection (**Figure 1**, **Table 2**)

**Table 2:** HBV infection patterns based on phylogenetic analysis (n = 42).

Pattern	n (%)	Median CD4+ (IQR)	ART interruption, n (%)
Reactivation	30 (71.4)	156 (98–212)	22 (73.3)
New infection	12 (28.6)	198 (134–267)	4 (33.3)

ART, antiretroviral therapy; IQR, interquartile range.

Patients classified as reactivation had lower median CD4+ counts (156 cells/µL [IQR 98–212]) compared with those with apparent new infection (198 cells/µL [IQR 134–267]). Reactivation was associated with documented antiretroviral therapy interruption in 73% (22/30) of cases.

### Cytokine profiles

**Figure 2:**
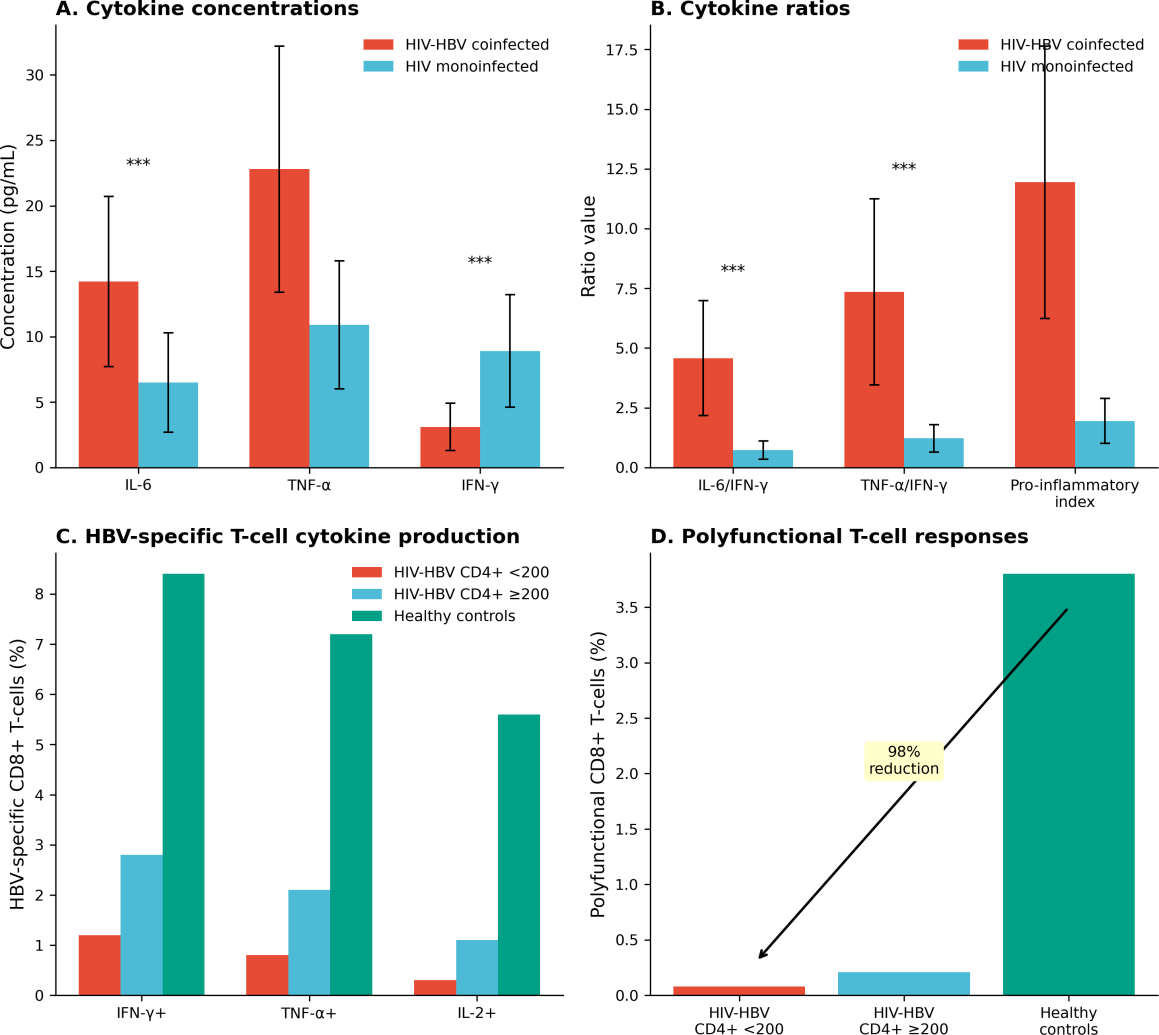
Cytokine dysregulation and T-cell dysfunction in HIV-HBV coinfection. **(A)** Plasma cytokine concentrations in coinfected vs monoinfected patients. **(B)** Cytokine ratios demonstrating pro-inflammatory predominance. **(C)** HBV-specific CD8+ T-cell responses stratified by CD4+ count. **(D)** Polyfunctional T-cell responses showing 98% reduction in coinfected patients vs controls. ***p < 0.001.

Coinfected patients demonstrated marked cytokine dysregulation compared with HIV-monoinfected controls (**Table 3**; **Figures 2A, B**). IL-6 concentrations were elevated in coinfection (median 14.2 pg/mL [IQR 8.7–21.8] vs 6.5 pg/mL [3.2–10.9]; p < 0.001), as were TNF-α levels (22.8 pg/mL [15.4–34.2] vs 10.9 pg/mL [6.8–16.7]; p < 0.001). Conversely, IFN-γ was reduced (3.1 pg/mL [1.8–5.4] vs 8.9 pg/mL [5.6–14.2]; p < 0.001).

**Table 3:** Cytokine profiles in HIV-HBV coinfected vs HIV monoinfected patients.

Parameter	Coinfected (n=59)	Monoinfected (n=59)	p-value
IL-6, pg/mL	14.2 (8.7–21.8)	6.5 (3.2–10.9)	<0.001
TNF-α, pg/mL	22.8 (15.4–34.2)	10.9 (6.8–16.7)	<0.001
IFN-γ, pg/mL	3.1 (1.8–5.4)	8.9 (5.6–14.2)	<0.001
IL-6/IFN-γ ratio	4.58 (2.84–7.62)	0.73 (0.42–1.18)	<0.001

Values are median (IQR). IL-6, interleukin-6; TNF-α, tumor necrosis factor-alpha; IFN-γ, interferon-gamma.

The IL-6/IFN-γ ratio was 6.3-fold higher in coinfected patients (median 4.58 vs 0.73; p < 0.001), reflecting the shift from antiviral to pro-inflammatory predominance. These findings indicate a shift toward a pro-inflammatory immune environment with reduced antiviral activity, which may contribute to ongoing viral replication and liver injury in coinfected patients.

### HBV-specific T-cell responses

HBV-specific CD8+ T-cell responses were profoundly impaired in coinfected patients (**Table 4**; **Figures 2C, D**). The proportion producing IFN-γ was markedly reduced (median 1.2% in patients with CD4+ <200 vs 8.4% in healthy controls; p < 0.001). Polyfunctional responses were nearly absent in severely immunosuppressed patients (0.08% vs 3.8% in controls; p < 0.001), representing a 98% reduction. The marked reduction in polyfunctional HBV-specific T-cell responses suggests impaired immune control of HBV, particularly in individuals with advanced immunosuppression.

**Table 4:** HBV-specific CD8+ T-cell responses.

Parameter	CD4+ <200 (n=25)	CD4+ ≥200 (n=34)	Controls (n=30)	p
IFN-γ+ cells, %	1.2 (0.6–2.1)	2.8 (1.4–4.2)	8.4 (6.1–11.2)	<0.001
Polyfunctional, %	0.08 (0.02–0.15)	0.21 (0.12–0.34)	3.8 (2.4–5.6)	<0.001
PD-1+, %	72.4 (64–81)	62.1 (54–71)	12.3 (8–18)	<0.001

Values are median (IQR). Polyfunctional: ≥2 cytokines. PD-1, programmed death-1.

PD-1 expression on HBV-specific CD8+ T-cells was elevated in coinfected patients (median 68.4% vs 12.3% in controls; p < 0.001), consistent with T-cell exhaustion.

### Correlations between immune parameters and clinical outcomes

**Figure 3:**
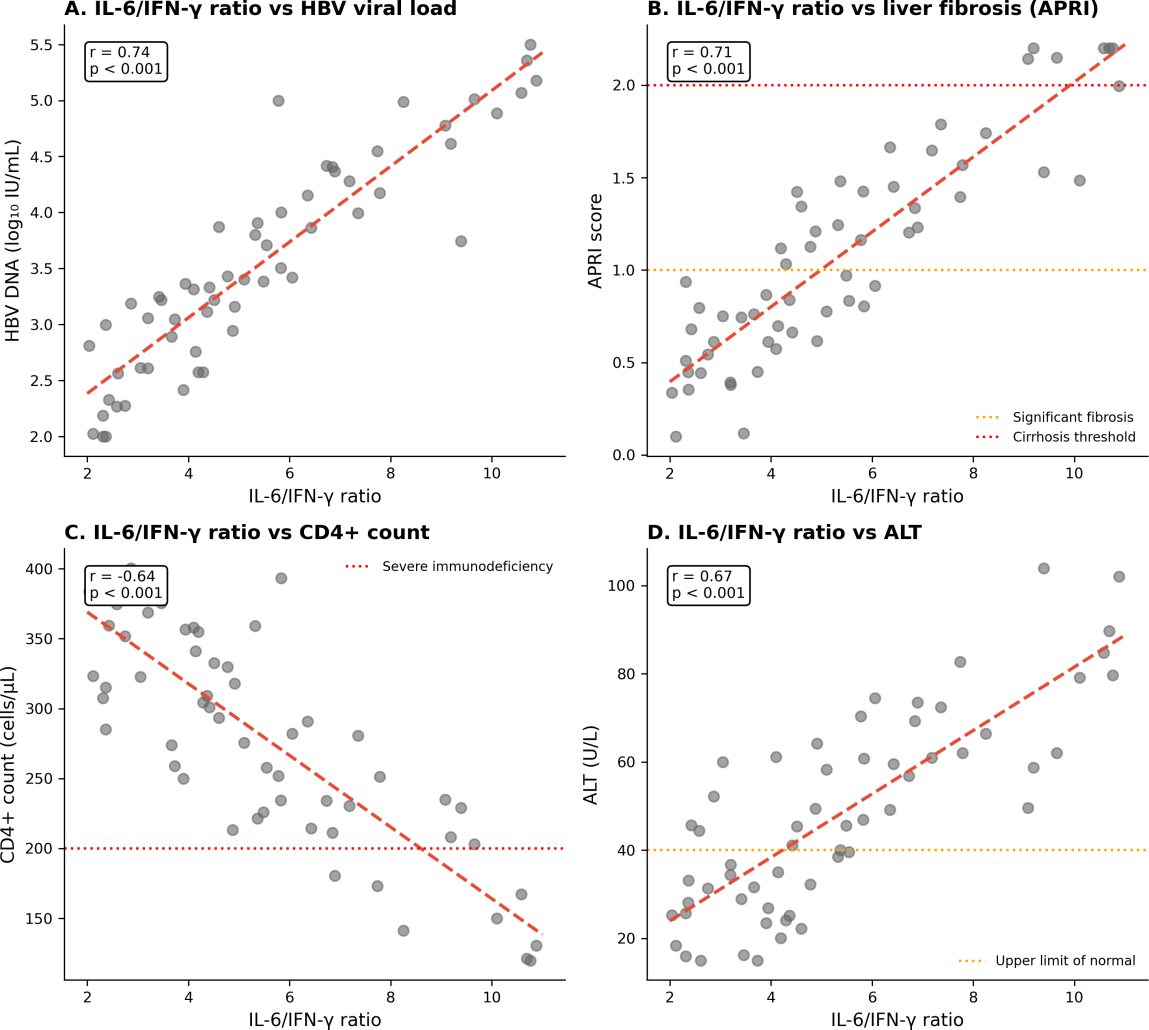
Correlations between IL-6/IFN-γ ratio and clinical parameters. **(A)** HBV viral load (r = 0.74). **(B)** APRI score (r = 0.71). **(C)** CD4+ count (r = −0.64). **(D)** ALT (r = 0.67). All p < 0.001.

The IL-6/IFN-γ ratio demonstrated strong correlations with clinical parameters (**Figure 3**). Higher ratios correlated with HBV DNA level viral load (r = 0.74, p < 0.001), APRI scores (r = 0.71, p < 0.001), and alanine aminotransferase (r = 0.67, p < 0.001), with an inverse correlation with CD4+ count (r = −0.64, p < 0.001).

### Liver fibrosis

Coinfected patients had elevated APRI scores compared with monoinfected controls (median 1.24 [IQR 0.68–2.14] vs 0.42 [0.28–0.62]; p < 0.001). Thirty-four participants (57.6%) had APRI >1.0, and 14 (23.7%) had scores >2.0, consistent with cirrhosis.

### Disease progression

**Figure 4:**
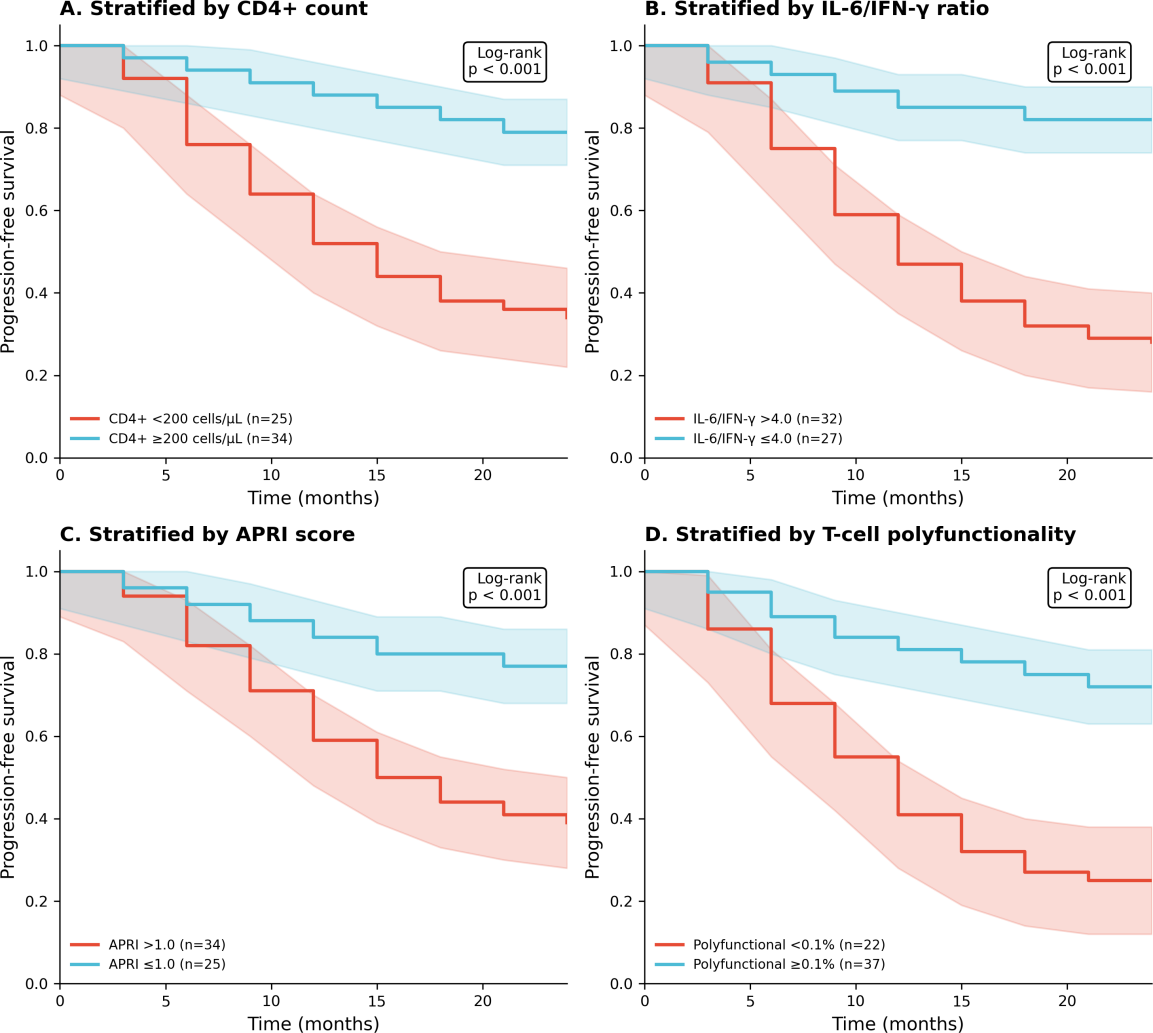
Kaplan-Meier progression-free survival curves stratified by **(A)** CD4+ count, **(B)** IL-6/IFN-γ ratio, **(C)** APRI score, and **(D)** T-cell polyfunctionality. All p < 0.001 by log-rank test.

During median follow-up of 18.4 months (IQR 12.6–22.8), 32 participants (54.2%) experienced the composite progression endpoint. Kaplan-Meier analysis demonstrated inferior progression-free survival in patients with CD4+ <200 cells/µL (24-month estimate 34.2% vs 78.6%; log-rank p < 0.001), IL-6/IFN-γ ratio >4.0 (28.4% vs 82.1%; p < 0.001), APRI >1.0 (38.6% vs 76.8%; p < 0.001), and polyfunctional T-cells <0.1% (24.6% vs 72.4%; p < 0.001) (**Figure 4**).

**Figure 5:**
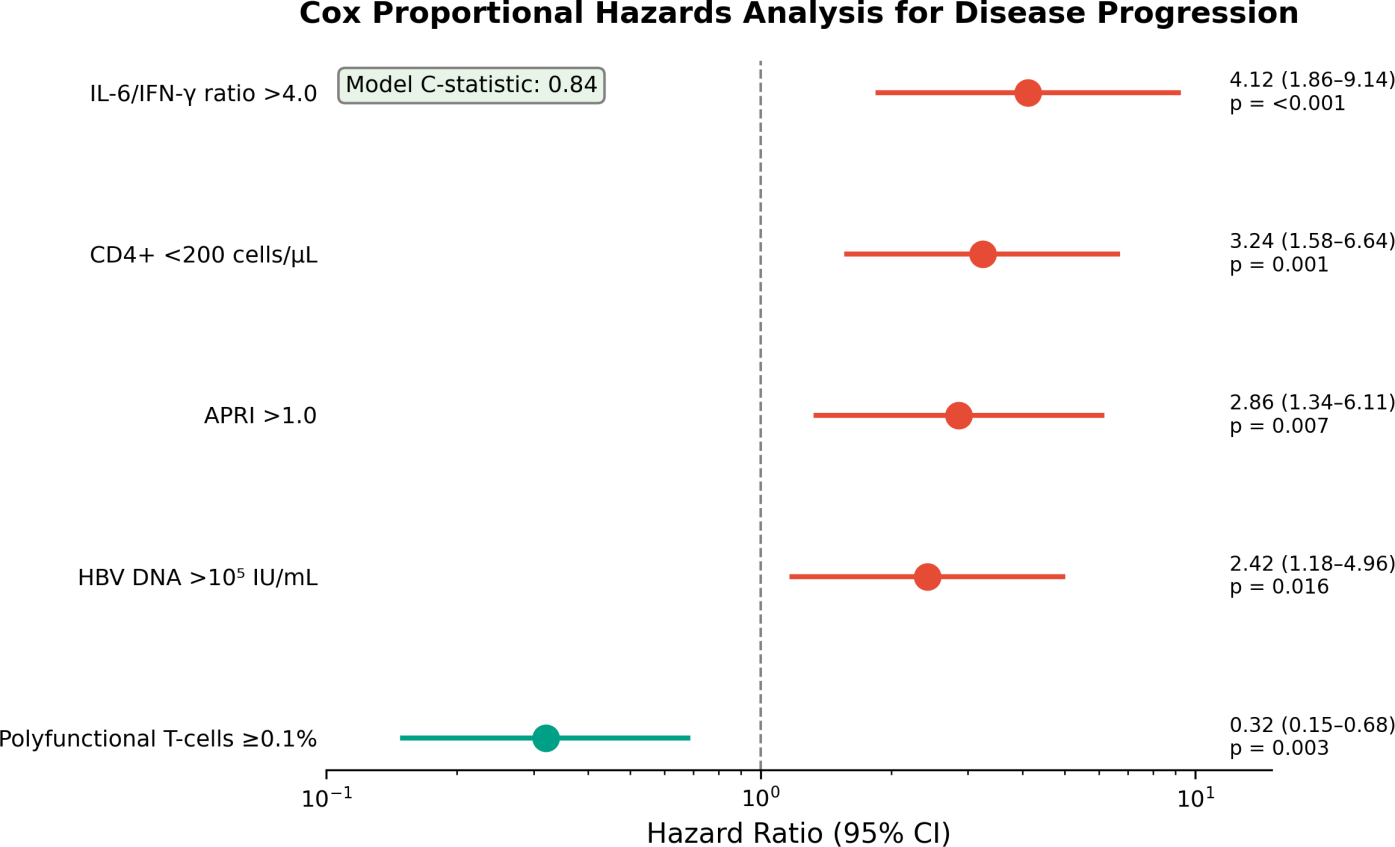
Forest plot of Cox proportional hazards analysis for disease progression. Model C-statistic: 0.84.

In multivariable Cox regression (**Table 5**, **Figure 5**), independent predictors of progression were IL-6/IFN-γ ratio >4.0 (HR 4.12, 95% CI 1.86–9.14; p < 0.001), CD4+ <200 cells/µL (HR 3.24, 95% CI 1.58–6.64; p = 0.001), APRI >1.0 (HR 2.86, 95% CI 1.34–6.11; p = 0.007), and HBV DNA (viral Load) >10^5^ IU/mL (HR 2.42, 95% CI 1.18–4.96; p = 0.016). Preserved T-cell polyfunctionality was protective (HR 0.32, 95% CI 0.15–0.68; p = 0.003). The model C-statistic was 0.84.

**Table 5:** Cox proportional hazards regression for disease progression.

Variable	HR (95% CI)	p-value
IL-6/IFN-γ ratio >4.0	4.12 (1.86–9.14)	<0.001
CD4+ <200 cells/µL	3.24 (1.58–6.64)	0.001
APRI >1.0	2.86 (1.34–6.11)	0.007
HBV DNA >10^5^ IU/mL	2.42 (1.18–4.96)	0.016
Polyfunctional T-cells ≥0.1%	0.32 (0.15–0.68)	0.003

HR, hazard ratio; CI, confidence interval. Model C-statistic: 0.84.

## Discussion

This prospective study demonstrates that HIV-HBV coinfection is characterized by HBV reactivation, cytokine imbalance favoring pro-inflammatory responses, and profound virus-specific T-cell dysfunction. These immunological and virological features were associated with liver fibrosis and disease progression.

Phylogenetic analysis indicated that most cases of rising HBV viraemia (71.4%) were consistent with reactivation rather than new infection acquisition. This finding has clinical implications: patients with prior HBV exposure remain at risk of viral rebound during immunosuppression, even with previously suppressed or low-level viraemia (9, 24). The strong association between reactivation and both low CD4+ counts and antiretroviral therapy interruption underscores the importance of sustained immune reconstitution for HBV control.

The cytokine profile observed—elevated IL-6 and TNF-α with reduced IFN-γ—indicates an immune environment promoting hepatic inflammation whilst impairing viral clearance (11, 12). IL-6 contributes to hepatic fibrogenesis and acute-phase responses, whilst TNF-α mediates hepatocyte injury (25, 26). The relative deficiency of IFN-γ, essential for non-cytolytic HBV clearance, may permit ongoing viral replication (27). The IL-6/IFN-γ ratio captured this imbalance effectively and consistently outperformed individual cytokines as a correlate of viral and clinical parameters.

The near-complete loss of HBV-specific polyfunctional T-cells in severely immunosuppressed patients indicates advanced immune exhaustion extending beyond numerical CD4+ depletion. Polyfunctional T-cells are associated with superior pathogen control (13, 28). Their absence, combined with elevated PD-1 expression, suggests durable dysfunction that may persist despite antiretroviral therapy (14, 29).

The high prevalence of significant fibrosis (57.6% with APRI >1.0) in this relatively young cohort underscores the accelerated hepatic injury in coinfection. The strong correlation between cytokine ratios and APRI scores supports a mechanistic link between immune dysregulation and fibrogenesis (30).

Several limitations warrant consideration. The cohort size, whilst adequate for the primary analyses, limits power for smaller subgroup comparisons. Fibrosis assessment relied on non-invasive indices rather than elastography or histology (31). Healthy controls for T-cell assays were recruited separately, potentially introducing residual confounding. The composite progression endpoint combined heterogeneous outcomes. Finally, the observational design precludes causal inference.

These findings have several practical clinical implications. First, the strong association between HBV DNA level, cytokine imbalance, and disease progression highlights the importance of routine viral load monitoring in coinfected patients, particularly those with low CD4+ counts. Second, the IL-6/IFN-γ ratio may serve as a simple adjunct marker to identify individuals at higher risk of fibrosis and clinical deterioration and warrants validation in independent cohorts (32). Third, the profound reduction in HBV-specific polyfunctional T-cell responses supports early initiation and strict adherence to tenofovir-containing antiretroviral regimens to maintain immune control and prevent reactivation (33–35). Longitudinal monitoring of immune and virological markers may therefore help guide closer clinical follow-up, earlier hepatology referral, and prioritization of patients for intensified treatment and surveillance strategies.

## Conclusions

HIV-HBV coinfection is characterized by HBV reactivation, cytokine imbalance, and virus-specific T-cell exhaustion. These immunological and virological parameters correlate with liver fibrosis and disease progression. The IL-6/IFN-γ ratio and T-cell polyfunctionality warrant further evaluation as markers for risk stratification in coinfected populations.

## Data Availability

The original contributions presented in the study are included in the article/**Supplementary Material**. Further inquiries can be directed to the corresponding author/s.
