# Supplementary figures and images for: Cytokine imbalance and HBV-specific T-cell exhaustion predict disease progression in HIV-HBV coinfection
Source: Front Immunol. 2026 Mar 4;17:1789692. doi: 10.3389/fimmu.2026.1789692 (PMC12996162; doi:10.3389/fimmu.2026.1789692)

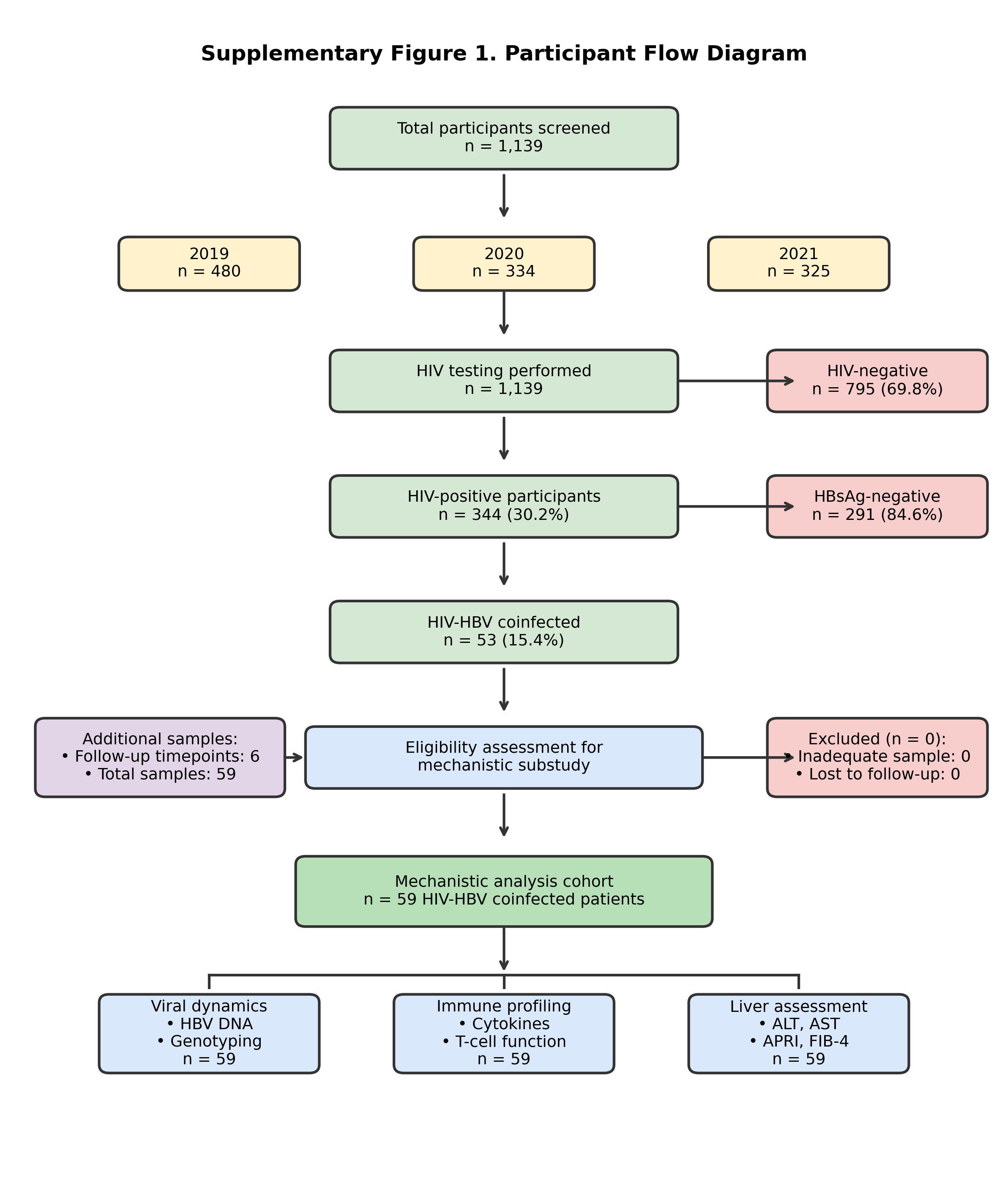

Supplement: Supplementary file 1 [file Image1.tiff]
