# Supplementary material for: Cytokine imbalance and HBV-specific T-cell exhaustion predict disease progression in HIV-HBV coinfection
Source: Front Immunol. 2026 Mar 4;17:1789692. doi: 10.3389/fimmu.2026.1789692 (PMC12996162; doi:10.3389/fimmu.2026.1789692)
Supplement: Supplementary file 2 [file Table1.docx]

**Table 6. HBV Reference Sequences Used for Phylogenetic Analysis**

| **Accession Number** | **Genotype** | **Geographic Origin** | **Description** |
| --- | --- | --- | --- |
| X75657 | E | West Africa | Prototype reference |
| AB091255 | E | Ghana | Clinical isolate |
| GQ161830 | E | Cameroon | Clinical isolate |
| FN545833 | E | Benin | Clinical isolate |
| HM363592 | E | Nigeria | Clinical isolate |
| X02763 | A | Global | Prototype reference |
| AB076679 | A | South Africa | Clinical isolate |
| X02496 | D | Global | Prototype reference (outgroup) |

*Reference sequences were obtained from GenBank (https://www.ncbi.nlm.nih.gov/genbank/).*
